# Rapid typing diagnosis and clinical analysis of subtypes A and B of human respiratory syncytial virus in children

**DOI:** 10.1186/s12985-022-01744-y

**Published:** 2022-01-21

**Authors:** Zheng Shen, Yuanyuan Zhang, Huamei Li, Lizhong Du

**Affiliations:** 1 Department of Clinical Laboratory, Zhejiang University School of Medicine Children’s Hospital, 3333 Binsheng Road, Hangzhou, 310051 Zhejiang China; 2 Department of Respiratory, Zhejiang University School of Medicine Children’s Hospital, 3333 Binsheng Road, Hangzhou, 310051 Zhejiang China; 3 Department of Neonatology, Zhejiang University School of Medicine Children’s Hospital, 3333 Binsheng Road, Hangzhou, 310051 Zhejiang China; 4 National Clinical Research Center for Child Health, 3333 Binsheng Road, Hangzhou, 310051 Zhejiang China

**Keywords:** Human respiratory syncytial virus, Children, Acute respiratory infections, Subtype

## Abstract

**Background:**

Human respiratory syncytial virus (HRSV) is the leading pathogens causing acute respiratory infections (ARI) in children under five years old. We aimed to investigate the distribution of HRSV subtypes and explore the relationship between viral subtypes and clinical symptoms and disease severity.

**Methods:**

From November 2016 to April 2017, 541 children hospitalized because of ARI were included in the study. Throat swabs were collected for analysis and all samples were tested by multiplex one-step qRT-PCR for quantitative analysis and typing of HRSV. Patients’ demographics, clinical symptoms as well as laboratory and imaging results were retrieved from medical records.

**Results:**

HRSV was detected in 19.6% of children hospitalized due to ARI. HRSV-positive children were younger (*P* < 0.001), had a higher frequency of wheezing and pulmonary rales (*P* < 0.001; *P* = 0.003), and were more likely to develop bronchopneumonia (*P* < 0.001). Interleukin (IL) 10、CD4/CD8 (below normal range) and C-reactive protein levels between subtypes A and B groups were significantly different (*P* = 0.037; *P* = 0.029; *P* = 0.007), and gender differences were evident. By age-stratified analysis between subtypes A and B, we found significant differences in fever frequency and lymphocyte ratio (*P* = 0.008; *P* = 0.03) in the 6–12 months age group, while the 12. 1–36 months age group showed significant differences in fever days and count of leukocytes, platelets, levels aspartate aminotransferase, IL-6, lactate dehydrogenase and proportion CD4 positive T cells(*P* = 0.013; *P* = 0.018; *P* = 0.016; *P* = 0.037; *P* = 0.049; *P* = 0.025; *P* = 0.04). We also found a positive correlation between viral load and wheezing days in subtype A (*P* < 0.05), and a negative correlation between age, monocyte percentage and LDH concentration in subtype B (*P* < 0.05).

**Conclusions:**

HRSV is the main causative virus of bronchopneumonia in infants and children. The multiplex one-step qRT-PCR not only provides a rapid and effective diagnosis of HRSV infection, but also allows its typing. There were no significant differences in the severity of HRSV infection between subtypes A and B, except significant gender-specific and age-specific differences in some clinical characteristics and laboratory results. Knowing the viral load of HRSV infection can help understanding the clinical features of different subtypes of HRSV infection.

## Introduction

Human respiratory syncytial virus (HRSV) is the most common pathogen causing acute lower respiratory tract infections (ALRI) in infants and young children [1–4], and upper respiratory tract infections in children [5–8]. Over 90% of children infected with HRSV are below two years of age and 0.5%–2% of patients will require hospitalization because of severe symptoms [9]. HRSV causes regular seasonal epidemics, usually in the winter in temperate countries or during the rainy season in tropical areas [10, 11]. In 2015, Shi et al. [12, 13] estimated that 33.1 million cases of HRSV-ALRI occurred in children under 5 years of age, resulting in approximately 3.2 million hospitalizations and 59,600 in-hospital deaths. Nearly 45% of hospital admissions and inpatient deaths occurred in children under 6 months of age. HRSV is a non-segmented single negative strand RNA enveloped virus belonging to the Paramyxoviridae family genus pneumoviridae [14]. HRSV genomic RNA has about 15,200 nucleotides in length containing 10 genes, expressing 11 proteins. Among them, surface glycoproteins F and G are the major antigens responsible for cell attachment and inducing immune response neutralization, respectively [15]. HRSV can be divided into subtypes A and B based on different antigenic and genetic characteristics [16, 17]. These two subtypes circulate independently in the human population, with subtype A being more prevalent [18]. There are conflicting reports on the relationships between viral subtypes and clinical severity. Some reports revealed no significant clinical differences in severity between subtypes A and B HRSV infection [19–21]; while other studies revealed that different subtypes of HRSV were associatedwith the clinical characteristics and severity of the disease [22–27]. Meanwhile, there is little information about the association between viral load of HRSV subtypes A or B and their clinical characteristics and disease severity.

Therefore, we aimed to detect HRSV infections, identify virus subtypes and analyze viral load of hospitalized children with ARI from November 2016 to April 2017 in Zhejiang Province by multiplex One-Step qRT-PCR. We also explored the correlation between different subtypes and viral load of HRSV and clinical characteristics as well as severity of the disease. This study will help improve the prevention, control and treatment of HRSV in Zhejiang Province, which can help to reduce both patient hospitalization time and its associated financial costs.

## Methods

### Subjects and clinical samples

This study included 541 hospitalized children with ARI, mainly presenting with bronchopneumonia or pneumonia, between November 2016 and April 2017. Patients’ demographics, clinical symptoms, and laboratory and imaging results were retrieved from medical records. There were 325 males and 216 females with an average age of 29.74 ± 1.50 months (range: 0.03–167 months). A total of 357 patients had capillary bronchopneumonia; 117 had pneumonia; 5 had upper respiratory tract infection. Patients admitted due to other basic cardiopulmonary diseases, such as congenital heart disease, bronchiolitis obliterans, pulmonary bullae, bronchiectasis, bronchopulmonary dysplasia and those who developed ARI symptoms in the hospital were excluded. In order to exclude nosocomial infection, nasal, nasopharyngeal or throat swabs should be collected within the first 24 h of admission and 3 days after symptom onset for further study.

### Diagnosis criteria

Patients were diagnosed with ARI carrying respiratory symptoms (such as cough, rhinorrea, nasal congestion, sneezing) and signs of lower respiratory tract involvement (respiratory distress, crackles, wheezing, or chest-X ray infiltrates). Patients were diagnosed with neumonia carrying crackles or respiratory distress and X-ray pneumonic infiltrates, and bronchopneumonia carrying wheezing or respiratory distress.

### Immunofluorescence assay (IFA) of HRSV

HRSV was detected by direct immunofluorescence assay with D^3^ Ultra™ DFA RSV Reagent (Quidel, USA) according to the manufacturer’s instructions.

### Reference strains and plasmid

The reference strains used in this study were Long strains for HRSV subtype A and CH1856 for HRSV subtype B. Both strains were purchased from the China Center for Type Culture Collection and have been stored in our Central Laboratory, Children's Hospital, Zhejiang University School of Medicine, for many years. The recombinant positive plasmid containing HRSV M gene was provided by Shanghai Sangon Biotech Co., Ltd.

### HRSV quantitative detection and typing by multiplex one-step qRT-PCR

According to the sequences of all kinds of HRSV strains recorded in GeneBank, the HRSV universal primers and probes targeting M gene, subtypes A and B primers and probes targeting F gene were designed by Primer Express software. The sequences of primers and probes (synthesized by TaKaRa, JPN) are shown in Table 1. Total RNA of HRSV was extracted from nasal, nasopharyngeal or throat swabs with 500 uL viral preservation fluid by Trizol (Invitrogen, USA) according to the manufacturer’s instructions. HRSV was quantitatively detected and typed using multiplex One-Step qRT-PCR according to the manufacturer’s instructions (TaKaRa, JPN). The amplification program including a reverse transcription step was performed on an ABI-7500 Real-Time PCR System (Applied Biosystems, USA) using a panel of oligonucleotide primers and dual labelled hydrolysis (Taqman®) probes under the following conditions: 50 °C for 15 min, 94 °C for 2 min and 40 cycles of 10 s at 94 °C, 40 s at 60 °C. No template negative and positive controls for all primer/probe sets were included in each run.

**Table 1:** Multiplex One-Step qRT-PCR primers and probes used for quantitative detection and typing of HRSV

Name	Sequence (5’-3’)	Size (bp)
HRSV-M up	GCAAATATGGAAACATACGTGAA	
HRSV-M down	ACCCATATTGTW(T/A)AGTGATGCAG	
HRSV-M probe	FAM-CTTCACGAR(A/G)GGCTCCACATACACAGC-BHQ1	113
HRSVA-F up	TTGGTTTTTTGTTAGGTGTTGGAT	
HRSVA-F down	GGCCTTGTTTGTGGATAGTAGA	
HRSVA-F probe	JOE-AATCGCCAGTGGCATTGCTGTATCTAAGGT-BHQ1	118
HRSVB-F up	ACAGCTACCTATCTATGG	
HRSVB-F down	GTGGAAAGAAGGATACTG	
HRSVB-F probe	TAMRA-TCACAATACCATCCTCTATCAGTCCTT-BHQ2	158

### Clinical testing

4–5 mL of peripheral venous blood was collected immediately from all patients after admissionfor blood routine, C-reactive protein (CRP), liver function, myocardial enzyme spectrum, calcitonin, cytokines and other laboratory examinations. HP Sysmex xs-800i was used to test blood routine and CRP. Beckman AU5800 (Beckman Kurt LTD, USA) automatic biochemical analyzer was used to determine liver function and myocardial enzyme spectrum.

Serum IL-2, IL-4, IL-6, IL-10, TNF-α and IFN-γ levels were determined using a CBA HumanTh1/Th2 Cytokine Kit II (BD Biosciences, San Diego, CA, USA) according to the manufacturer's specifications. Following the acquisition of sample data using a FACScalibur flow cytometer (BD Biosciences), results were generated using BD CBA Software (BD Biosciences, San Jose, CA, USA). Then we established the standard curve for each reagent. 1.0 pg/mL was the lowest detection limit for these six cytokines, while 5000 pg/mL was the highest.

T-cell subsets were detected by multicolor flow cytometry (FACSCalibur, BD, USA) using blood samples containing heparin. Mouse anti-human CD45-FITC, CD3-PC5, CD4-PE, and CD8-ECD monoclonal antibodies, and other reagents were purchased from BD. Data was analyzed by the MultiTEST software.

### Statistical analysis

The Kolmogorov–Smirnov normality test was used to determine if the data were normally distributed. Continuous normally distributed data were analyzed by the independent-Samples *t*-test. The Mann–Whitney *U* test was used to compare the differences in nonparametric variables (non-normally distributed data). Categorical variables were presented as proportion and analyzed by Pearson *Χ*^2^ or Fisher’s exact test. Results were expressed as *n* (%) or median (interquartile range). Kendall correlation analysis was used for grading variable data, whereas Spearman correlation analysis was used for continuous variable data. All statistical analyses were performed using SPSS version 22.0. A two-tailed test should be indicated for the *P* value. *P* < 0.05 was considered to be statistically significant.

### Ethics statement

The study was carried out at our third-level grade A hospital which provides specialized pediatric medical care to Zhejiang Province. The hospital has 1200 pediatric beds, as well as 200 neonatal unit beds and 50 intensive care beds. This study was approved by the Medical Ethical Committee of the Children Hospital of Zhejiang University School of Medicine (2021-IRB-011) and informed consent was obtained from all parents and/or legal guardian of participating children. The study complied with the Declaration of Helsinki.

## Results

### The characteristics of children with HRSV

**Fig. 1:**
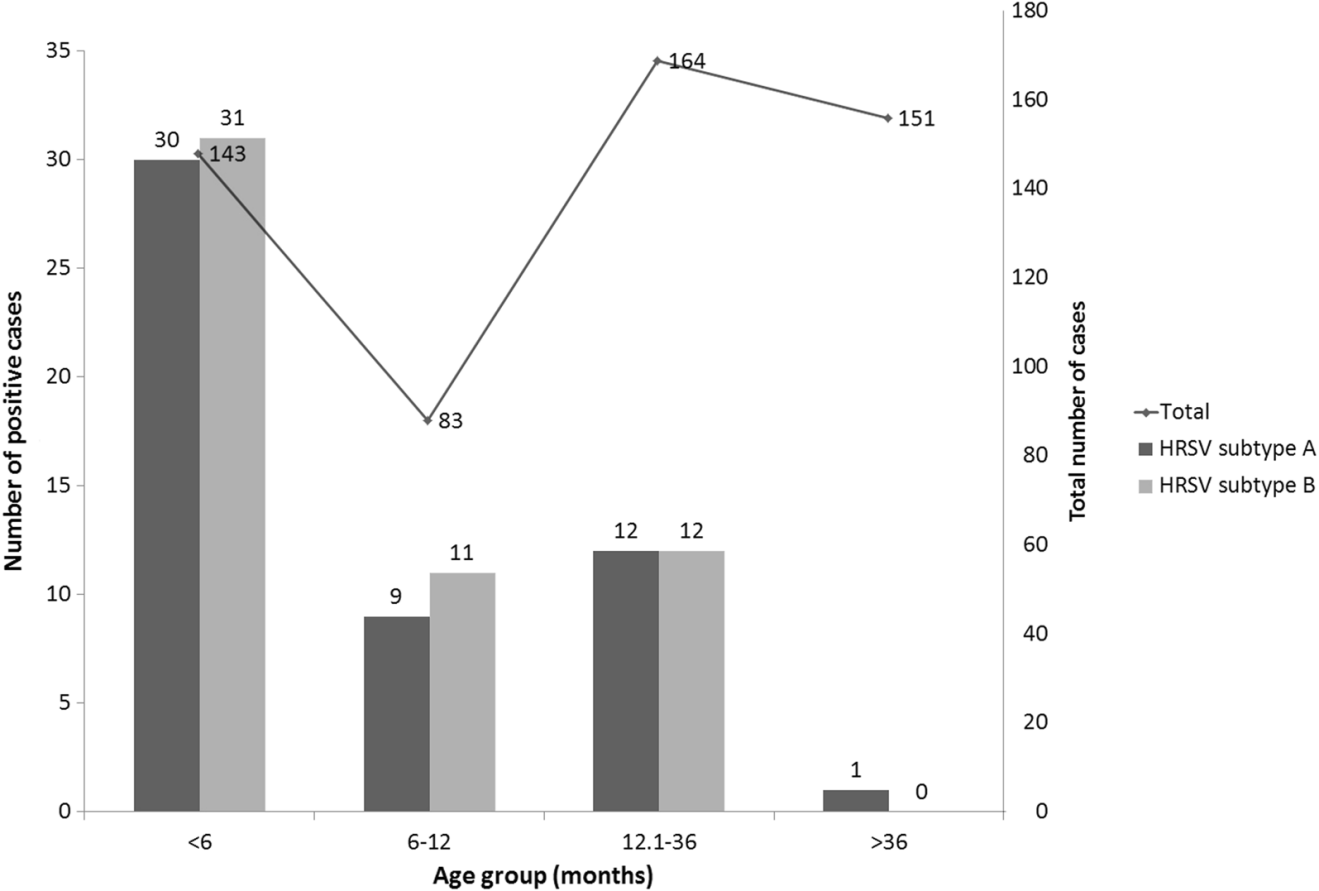
Distribution of cases of human respiratory syncytial virus (HRSV) subtypes A and B infection by age group

Of 541 nasopharyngeal aspirate samples from patients admitted to our hospital with ARI, 106 were infected with HRSV. HRSV-positive patients were generally younger than those not infected with HRSV (5.2 months vs. 18 months; *P* < 0.001). Infants less than 6 months of age showed the highest frequency (42.7%) of HRSV-positive cases. The frequency of HRSV-positive cases remained high in infants between 6 and 12 months of age (24.1%), and tended to decrease in older children (Fig. 1). There were differences in clinical symptoms between children infected and those not infected with HRSV. The rate of wheezing and pulmonary rales were higher in HRSV-positive children than that in HRSV-negative children (64.2% vs. 37.5%, P < 0.001; 59.4% vs. 43.2%, P = 0.003). Children infected with HRSV were more likely to develop bronchopneumonia than uninfected children (84.9% vs. 62.1%, *P* < 0.001) but significantly less likely to develop pneumonia or other diseases (Table 2).

**Table 2:** The characteristics of children with and without HRSV

Variable	HRSV positive (N = 106)	HRSV negative (N = 435)	P value
Sex^a^			P = 0.070
Male (%)	73 (22.1)	258 (77.9)	
Female (%)	33 (15.7)	177 (84.3)	
Age, months, median (IQR) ^b^	5.2(1.07–41)	18(0.03–167)	**P < 0.001**
< 6 ^b^	2.47(1.07-.587)	3.42(0.03–5.97)	P = 0.283
6–12 ^b^	9.84(6.3–12)	9.17(6.1–12)	P = 0.628
12.1–36 ^b^	17(12.07–35)	18(12.2–36)	P = 0.090
> 36 ^b^	41(41–41)	70(36.33–167)	P = 0.265
Clinical characteristics			
Fever, (%)T ≥ 37 .5 °C ^a^	33(31.1)	167(38.4)	P = 0.165
Cough, No. (%)^a^	104(97.1)	409(94.0)	P = 0.088
Wheezing, No. (%)^a^	68(64.2)	163(37.5)	**P < 0.001**
Rales, No. (%)^a^	63(59.4)	188(43.2)	**P = 0.003**
Respiratory rate, breaths/minute, No. (%)^a^	67(63.2)	309(71.0)	P = 0.117
Heart rate/minute, No. (%)^a^	78(73.6)	350(80.5)	P = 0.118
SpO_**2**_, No. (%)^a^	32/102(31.4)	157/427(36.8)	P = 0.307
Final diagnosis			
Bronchopneumonia, No. (%)^a^	90(84.9)	270(62.1)	**P < 0.001**
Pneumonia, No. (%)^a^	14(13.2)	105(24.2)	**P = 0.015**
Pertussis syndrome, No. (%)^a^	0(0)	5(1.1)	P = 0.589
Upper respiratory-tract infection, No. (%)^a^	0(0)	5(1.1)	P = 0.589
Others, No. (%)^a^	2(1.9)	50(11.5)	**P = 0.003**
Hospitalization duration (days) (median, IQR) ^b^	6(1–15)	6(1–67)	P = 0.985

IQR, interquartile range; HRSV, human respiratory syncytial virus; SpO_2_, oxygen saturation

Results are expressed as n (%) or median (IQR)

^a^P values by Pearson χ^2^ test

^b^Median and IQR, P values by Mann–Whitney U test

P < 0.05 in univariate analysis, proportions compared with *Χ*^*2*^or Fisher’s exact tests and continuous variables compared with the Wilcoxon rank sum test

### HRSV identification and typing

All nasopharyngeal aspirates samples were analyzed by IFA and multiplex One-Step qRT-PCR. IFA results showed that 61 samples were HRSV positive, with an HRSV infection rate of 11.3%. Multiplex One-Step qRT-PCR showed that 106 samples (19.6%) were positive for HRSV infection. So the sensitivity of multiplex One-Step qRT-PCR was significantly higher than that of IFA (*Χ*^*2*^ = 207.356; *P* < 0.001) (Table 3). At the same time, the 106 HRSV-positive samples were subtyped by multiplex One-Step qRT-PCR, with 52(49.1%) HRSV subtype A viruses and 54(50.9%) HRSV subtype B viruses. There was no significant difference in the rates for subtypes A and B HRSV infection.

**Table 3:** The comparison of IFA and multiplex One-Step qRT-PCR of HRSV detection

			IFA		Total
			Negative	Positive	
qRT-PCR	Negative	Count Percentage	428 79.1%	7 1.3%	435 80.4%
	Positive	Count Percentage	52 9.6%	54 10.0%	106 19.6%
Total	Count Percentage		480 88.7%	61 11.3%	541 100%

Pearson *Χ*^*2*^ = 207.356; P < 0.001

### Clinical and laboratory characteristics of HRSV subtypes A and B

**Fig. 2:**
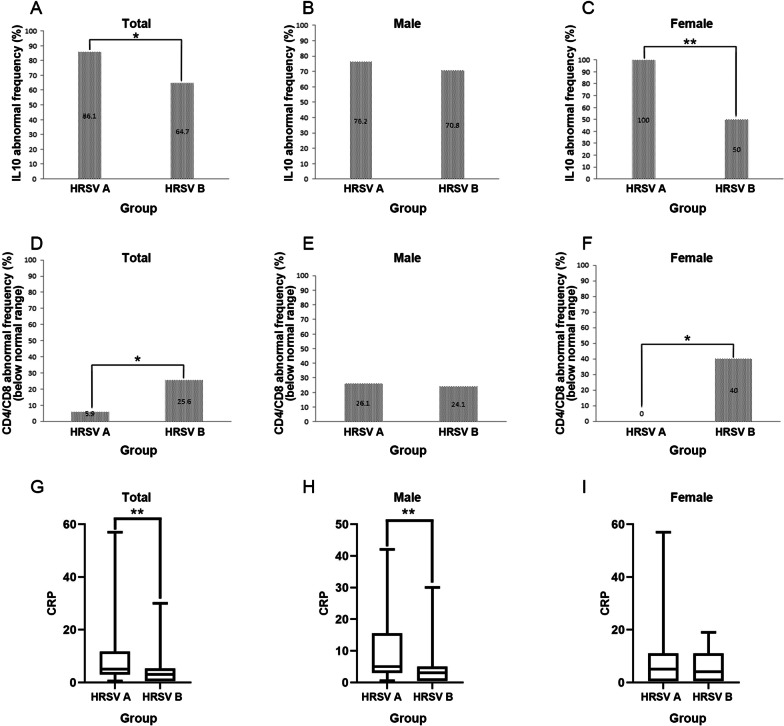
HRSV subtypes A and B infection cause changes in related indicators. **a**–**c** The changes of IL10 abnormal frequency in overall, male and female; **d**–**f** The changes of CD4/CD8 abnormal frequency (below normal range) in overall, male and female; **g**–**i** The changes of CRP in overall, male and female

Among 106 patients infected with HRSV, 52 were infected with HRSV subtype A and 54 were infected with HRSV subtype B. IL-10 and CD4/CD8 (below normal range) levels between subtypes A and B carriers were significantly different (*P* = 0.037; *P* = 0.029 respectively) (Fig. 2a, d). CRP was higher in patients infected with subtypes A than B (*P* = 0.007) (Fig. 2g). Further gender-stratified analysis showed a significantly higher frequency of IL-10 abnormalities in subtype A carriers than subtype B (*P* = 0.005) (Fig. 2c) and the CD4/CD8 (below normal range) abnormal frequency of subtype A carriers was significantly lower than that of subtype B (*P* = 0.035) (Fig. 2f) in female patients, but no significant difference in male patients (Fig. 2b, e). In contrast, male patients with subtype A had a significantly higher CRP level than those with subtype B (*P* = 0.008) (Fig. 2h), while there was no significant difference in female patients (Fig. 2i). Also, the frequency of abnormal CD4 count (below the normal range) was significantly lower in female patients infected with subtype A than those with subtype B (*P* = 0.012) (Table 4).

**Table 4:** The clinical and laboratory characteristics in the 106 patients grouped by HRSV subtype

Variable	HRSV-A (N = 52)	HRSV-B (N = 54)	P value
Clinical characteristics			
Sex^a^			P = 0.330
Male No. (%)	34(45.9)	40(54.1)	
Female No. (%)	18(56.3)	14(43.7)	
Age, months, median (IQR)^b^	5.23(1.1–41)	5.17(1.07–21)	P = 0.700
< 6 ^b^	2.77(1.1–5.87)	2.45(1.07–5.67)	P = 0.965
6–12 ^b^	10.87(6.63–11.1)	8.53(6.3–12)	P = 0.503
12.1–36^c^	20(12.47–35)	15.5(12.07–21)	P = 0.051
Clinical data			
Fever, No. (%) T ≥ 37.5°C^a^	20(38.5)	13(24.1)	P = 0.110
Fever, days, median (IQR)^b^	2(0–11)	0(0–8)	P = 0.056
Cough, No. (%)^a^	50(96.2)	54(100)	P = 0.238
Wheezing, No. (%)^a^	34(65.4)	34(63.0)	P = 0.795
Rales, No. (%)^a^	32(61.5)	31(57.4)	P = 0.665
Respiratory rate, breaths/minute, No. (%)^a^	35(67.3)	33(61.1)	P = 0.506
Heart rate/minute, No. (%)^a^	37(71.2)	42(77.8)	P = 0.434
SpO_2_, No. (%)^a^	15(28.8)	17/50(34)	P = 0.575
Final diagnosis			
Bronchopneumonia, No. (%)^a^	44(84.6)	46(85.1)	P = 0.935
Pneumonia, No. (%)^a^	7(13.5)	7(13.0)	P = 0.940
Others, No. (%)^a^	1(1.9)	1(1.9)	P = 1.000
Hospitalization duration, days, (median, IQR)^b^	6(3–12)	6.5(1–15)	P = 0.478
Laboratory results			
Hematological analysis			
WBC, × 10^9^/L, median (IQR) ^b^	8.4(3–22.8)	8.83(2.25–17.74)	P = 0.378
Lymphocyte%, median (IQR) ^b^	56.5(11.8–80.3)	59.9(9.6–85.5)	P = 0.303
Neutrophils%, median (IQR) ^b^	34.7(9.9–80.5)	29.65(11.1–84.3)	P = 0.310
Monocyte%, median (IQR)^c^	7.25(2.2–15.4)	8.3(0.7–18.2)	P = 0.497
Acidophil%, median (IQR)^b^	0.65(0.0–12.3)	0.8(0.0–10.4)	P = 0.487
Basophil%, median (IQR)^b^	0.3(0.0–1.2)	0.3(0.0–0.7)	P = 0.993
PLT, × 10^9^/L, median (IQR) ^b^	372.5(27.0–803.0)	372.0(166.0–753.0)	P = 0.507
Hemoglobin, median (IQR) ^b^	111.5(90.0–130.0)	117.0(78.0–149.0)	P = 0.108
CRP, mg/mL, median (IQR)^b^	5(0.5–57)	3(0.5–30)	**P = 0.007**
Male, median (IQR)^b^	5(0.5–42)	3(0.5–30)	**P = 0.008**
Female, median (IQR)^b^	5(0.5–57)	4(0.5–19)	P = 0.529
Cytokines data			
IL2, No. (%)^a^	0/36(0)	1/34(2.9)	P = 0.486
IL4, No. (%)^a^	25/36(69.4)	26/34(76.5)	P = 0.509
IL6, No. (%)^a^	10/36(27.8)	5/34(14.7)	P = 0.183
IL10, No. (%)^a^	31/36(86.1)	22/34(64.7)	**P = 0.037**
Male, No. (%)^a^	16/21(76.2)	17/24(70.8)	P = 0.685
Female, No. (%)^a^	15/15(100)	5/10(50)	**P = 0.005**
TNF, No. (%)^a^	0/36(0)	0/34(0)	**–**
INF-ɣ, No. (%)^a^	4/36(11.1)	3/34(8.8)	P = 1.000
T-cell subsets data			
CD20, below normal range, No. (%)^a^	16/32(50)	12/33(36.4)	P = 0.267
Above normal range, No. (%)^a^	8/32(25)	13/33(39.4)	P = 0.215
CD3, below normal range, No. (%)^a^	9/34(26.5)	14/39(35.9)	P = 0.387
Above normal range, No. (%)^a^	13/34(38.2)	14/39(35.9)	P = 0.836
CD4, below normal range, No. (%)^a^	6/34(17.6)	12/39(30.8)	P = 0.194
Male, No. (%)^a^	6/23(26.1)	7/29(24.1)	P = 0.872
Female, No. (%)^a^	0/11(0)	5/10(50)	**P = 0.012**
Above normal range, No. (%)^a^	20/34(58.8)	21/39(53.8)	P = 0.669
CD8, below normal range, No. (%)^a^	12/34(35.3)	13/39(33.3)	P = 0.860
Above normal range, No. (%)^a^	7/34(20.6)	10/39(25.6)	P = 0.610
CD3 − CD16 + CD56 + , below normal range, No. (%)^a^	20/31(64.5)	21/32(65.6)	P = 0.926
Above normal range, No. (%)^a^	0/31(0)	1/32(3.1)	P = 1.000
CD4/CD8, below normal range, No. (%)^a^	2/34(5.9)	10/39(25.6)	**P = 0.029**
Male, No. (%)^a^	2/23(26.1)	6/29(24.1)	P = 0.278
Female, No. (%)^a^	0/11(0)	4/10(40)	**P = 0.035**
Above normal range, No. (%)^a^	16/34(47.1)	15/39(38.5)	P = 0.459
PCT, No. (%)^a^	7/43(16.3)	1/42(2.4)	P = 0.058
Male, No. (%)^a^	3/24(12.5)	0/31(0)	P = 0.077
Female, No. (%)^a^	4/19(21.1)	1/11(9.1)	P = 0.626
Biochemical analysis			
AST, No. (%)^a^	14(26.9)	11(20.4)	P = 0.427
ALT, No. (%)^a^	6(11.5)	6(11.1)	P = 0.945
CK, No. (%)^a^	5(9.6)	3(5.6)	P = 0.484
CK-MB, No. (%)^a^	32(61.5)	33(61.1)	P = 0.964
LDH, No. (%)^a^	25(48.1)	20(37.0)	P = 0.250

WBC, white blood cell; PLT, platelet; CRP, C-reactive protein; IL2, Interleukin 2; IL4, Interleukin 4; IL6, Interleukin 6; IL10, Interleukin 10; TNF, tumor Necrosis Factor; INF-ɣ, interferon ɣ; PCT, procalcitonin; AST, aspartate aminotransferase; ALT, alanine aminotransferase; CK, creatine kinase; CK-MB, creatine kinase-MB activity; LDH, lactate dehydrogenase

Results are expressed as n (%) or median (IQR)

^a^P values by Pearson χ^2^ test

^b^Median and IQR, P values by Mann–Whitney U test

^c^Median and IQR, P values by Independent-Samples t test

P < 0.05 in univariate analysis, proportions compared with *Χ*^*2*^or Fisher’s exact tests and continuous variables compared with the Wilcoxon rank sum test

**Fig. 3:**
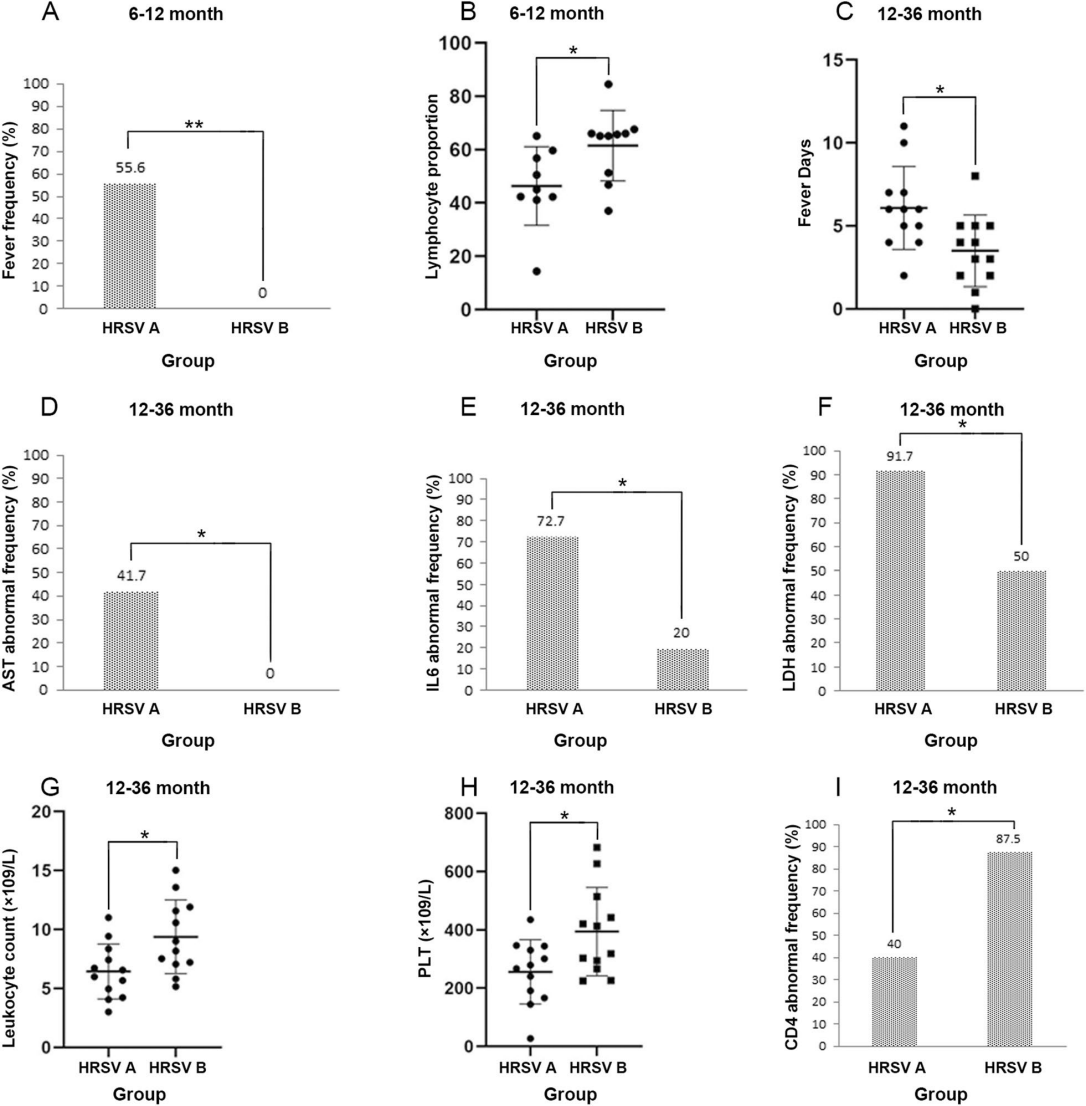
HRSV subtypes A and B infections in different age groups caused changes in related indicators. **a** Differences in the rate of the number of fever people in the 6–12 months age group; **b** Differences in the proportion of lymphocyte in the 6–12 months age group; **c** Differences in the number of fever days in the 12–36 months age group; 3D: Differences in AST abnormality rates in the 12–36 month age group; **e** Differences in IL6 abnormality rates in the 12–36 month age group; **f** Differences in LDH abnormality rates in the 12–36 month age group; **g** Differences in lymphocyte counts in the 12–36 month age group; **h** Differences in PLT counts between 12–36 months of age; **i** Differences in CD4 abnormality rates in the 12–36 month age group

We stratified 52 subtypes A patients and 54 B patients by age and found that children infected with subtype A had higher frequency of fever than those with subtype B (*P* = 0.008) (Fig. 3a). The proportion of lymphocytes in patients infected with subtype B was significantly higher than those with subtype A in the 6–12 months of age group (*P* = 0.03) (Fig. 3b). In the 12.1–36 months of age group, fever duration and the frequency of abnormal AST, IL-6 and LDH level in patients infected with subtype A were significantly higher than those with subtype B (*P* = 0.013; *P* = 0.037; *P* = 0.049; *P* = 0.025) (Fig. 3c–f), while the leukocyte count, platelet (PLT) and the frequency of abnormal CD4 were significantly lower than those infected with subtype B (*P* = 0.018; *P* = 0.016; *P* = 0.04). (Fig. 3g–i; Table 5).

**Table 5:** Clinical and laboratory characteristics of age stratification in 106 patients with HRSV subtype

Age stratification	Variable	HRSV-A (N = 9)	HRSV-B (N = 11)	P value
6–12 months	Fever, No. (%) T ≥ 37 .5°C^a^	5(55.6)	0(0)	P = 0.008
	Lymphocyte proportion, median (IQR)^c^	43.7(14.4–65.1)	65.7(37.0–84.5)	P = 0.030
Age stratification	Variable	HRSV-A (N = 12)	HRSV-B (N = 12)	P value
12.1–36 months	Fever, days,, median (IQR)^c^	6(2–11)	3.5(0–8)	P = 0.013
	WBC, × 10^9^/L, median (IQR)^c^	8.4(3–22.8)	8.83(2.25–17.74)	P = 0.018
	PLT, × 10^9^/L, median (IQR)^c^	259.5(27.0–435.0)	413.0(226.0–683.0)	P = 0.016
	AST, No. (%)^a^	5(41.7)	0(0)	P = 0.037
	IL6, No. (%)^a^	8(72.7)	1(20)	P = 0.049
	CD4, No. (%)^a^	4(40)	7(87.5)	P = 0.040
	LDH, No. (%)^a^	11(91.7)	6(50)	P = 0.025

Results are expressed as n (%) or median (IQR)

^a^P values by Pearson χ^2^ test

^b^Median and IQR, P values by Mann–Whitney U test

^c^Median and IQR, P values by Independent-Samples t test

P < 0.05 in univariate analysis, proportions compared with *Χ*^*2*^or Fisher’s exact tests and continuous variables compared with the Wilcoxon rank sum test

### Correlation of viral load with HRSV subtype A or B

In the correlation analysis between viral load and other variables, we found a positive correlation between viral load and wheezing days only in subtype A (*P* < 0.05). In contrast, in subtype B, viral load was negatively correlated with age, monocyte percentage and LDH (*P* < 0.05) (Table 6).

**Table 6:** Correlation between viral load and other variables in children with subtypes A and B

Variable	HRSV-A (N = 52)		HRSV-B (N = 54)	
	tau-b	P value	tau-b	P value
Age (month)	− 0.184	0.164	− **0.242**	**0.029**
Fever (day)^a^	− 0.118	0.339	− 0.175	0.154
Temperature	0.018	0.891	− 0.055	0.676
Rale	0.115	0.407	− 0.078	0.559
Cough (day)	0.069	0.556	0.038	0.736
Wheezing (day)	**0.275**	**0.023**	0.056	0.632
Respiratory rate/min	− 0.055	0.693	− 0.003	0.984
Heart rate/min	− 0.067	0.630	− 0.122	0.363
SpO_2_	0.035	0.800	0.039	0.779
Hospitalization duration (day)	0.197	0.105	0.069	0.548
WBC (10^9^/L)	− 0.169	0.223	− 0.049	0.716
Lymphocyte%	0.028	0.838	0.045	0.747
Neutrophils%	− 0.013	0.926	0.125	0.358
Monocyte%	− 0.213	0.159	− **0.305**	**0.049**
Acidophil%	− 0.012	0.939	0.110	0.482
Basophil%	− 0.103	0.497	− 0.157	0.261
PLT(10^9^/L)	− 0.093	0.501	0.156	0.243
Hemoglobin (g/L)	− 0.086	0.533	− 0.068	0.609
CRP (mg/mL)	− 0.080	0.562	− 0.043	0.749
IL2 (pg/ml)	− 0.031	0.827	0.301	0.074
IL4 (pg/ml)	0.085	0.614	− 0.004	0.982
IL6 (pg/ml)	− 0.044	0.795	− 0.079	0.640
IL10 (pg/ml)	0.284	0.093	0.299	0.076
TNF (pg/ml)	− 0.197	0.172	− 0.065	0.648
INF-ɣ (pg/ml)	− 0.250	0.139	0.284	0.092
CD20	0.234	0.187	− 0.052	0.760
CD3	0.087	0.611	− 0.174	0.266
CD4	0.156	0.364	− 0.068	0.665
CD8	− 0.056	0.744	0.199	0.204
CD3 − CD16 + CD56 +	0.228	0.206	0.000	1.000
CD4/CD8	− 0.126	0.465	0.289	0.065
PCT (ng/ml)	− 0.099	0.522	0.071	0.640
AST (U/L)	− 0.080	0.562	− 0.099	0.458
ALT (U/L)	− 0.149	0.282	− 0.169	0.205
CK (U/L)	0.198	0.154	0.122	0.361
CK-MB (U/L)	− 0.153	0.272	− 0.022	0.870
LDH (U/L)	− 0.179	0.198	− **0.441**	**0.001**

Kendall correlation analysis was used

## Discussion

HRSV is the most common viral pathogen causing ARI in infants, children, the elderly and high-risk populations. We first investigated the frequency of HRSV and its two subtypes, A and B, in children with ARI in Zhejiang Province and theirs correlation with clinical features and disease severity. We found that HRSV-positive children were younger, had a higher frequency of wheezing and pulmonary rales, and were more likely to develop bronchopneumonia. A positive correlation was found between viral load and wheezing days in patients infected with subtype A, and a negative correlation between age, monocyte percentage and LDH in patients with subtype B.

To investigate HRSV molecular epidemic characteristics in Zhejiang Province, we performed HRSV testing on 541 samples using both IFA and multiplex One-Step qRT-PCR. The results showed that the positive detection rate of IFA was significantly lower than that of multiplex One-Step qRT-PCR, which is similar to the findings of most studies [28–30]. The application of multiplex One-Step qRT-PCR indicates that molecular diagnostic techniques are more sensitive and can greatly improve the detection rate of HRSV, and being more efficient in identifying HRSV subtypes at the same time as diagnosis. However, multiplex One-Step qRT-PCR also has some limitations. qRT-PCR needs high-quality nucleic acid templates. Multiplex One-Step qRT-PCR will produce negative results when the nucleic acid template is broken, protein adhesion occurs, the content concentration is very low, or there are active ingredients in the nucleic acid template solution that inhibit Taq enzyme. Therefore, in this study, 7 samples were negative in multiplex One-Step qRT-PCR but positive in IFA. However, multiplex One-Step qRT-PCR cannot be denied because this method has good detection capabilities. The prevalence pattern of HRSV subtypes varies by region. In a given year, subtypes A and B may co-exist in a region or one of them may be predominant. In this study, out of the total 106 HRSV cases, 52 were subtype A and 54 were subtype B. This indicated that both subtypes were co-circulating in Zhejiang Province from November 2016 to April 2017. No significant difference in the predominance of the subtypes A and B was found, which were in agreement with the findings of some studies on the epidemiology of HRSV subtypes [31–33].

**Fig. 4:**
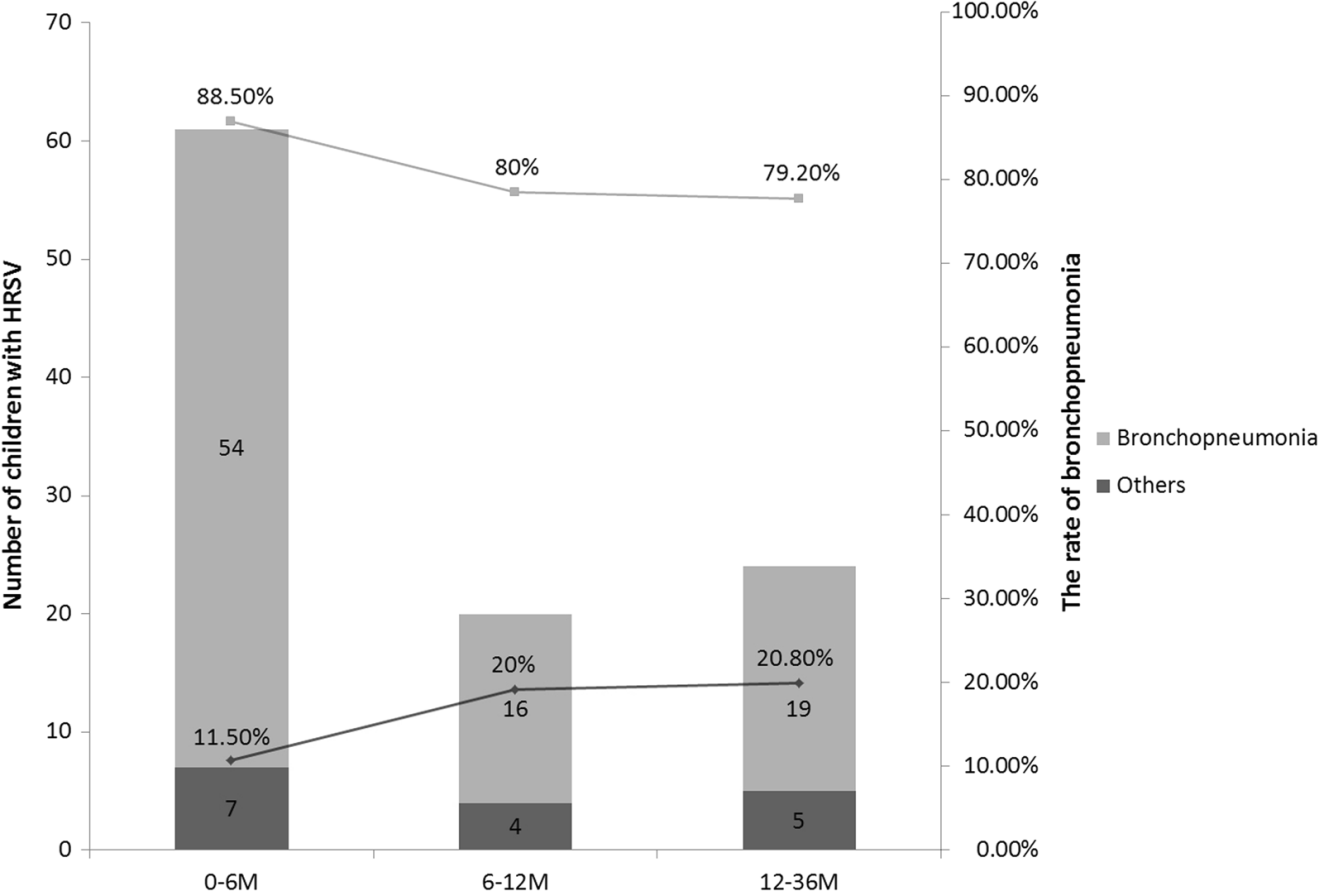
Distribution of bronchopneumonia of human respiratory syncytial virus (HRSV) infection by age group

In this study, we found that most infected individuals were infants aged 0–6 months, accounting for 57.5% of all samples, which were highly consistent with other studies form other countries or regions [34, 35]. Through the analysis of clinical manifestations, we found children infected with HRSV had a significantly higher rate of bronchopneumonia than those with other respiratory infections, and that all patients who developed capillary bronchopneumonia were infected with HRSV. In contrast, children infected with HRSV had a lower rate of pneumonia than those with other respiratory infections. The results suggest that HRSV is the most common pathogen in bronchopneumonia infections in children, especially in capillary bronchopneumonia infections, which is similar with Manjarrez-Zavala ME et al.’s study [36]. It may also be the reason why HRSV-infected children are more likely to develop wheezing and pulmonary rales. Similar with other studies [37–40], we also found that the rate of bronchopneumonia among children with HRSV infection decreased with age, suggesting that children with HRSV infection are more likely to develop bronchopneumonia at younger ages (Fig. 4).

The study of the relationship between viral subtypes (usually based on subtypes A and B) and disease severity has been the focus of many studies. Some studies have found that disease severity may vary with HRSV subtypes [41]. However, others have found conflicting results. They concluded that there was no significant difference in the clinical severity of subtypes A or B infection [19–21, 42]. In our study, we tested the hypothesis that disease severity may differ between HRSV of different subtypes. First, we found that the mean age of patients infected with subtype A was higher than those infected with subtype B, but without significant difference. The results after age grouping showed no significant difference in the prevalence of infection in each age group for subtypes A and B. Second, the clinical characteristics of patients infected with subtypes A and B HRSV infections were analyzed. We found no significant difference between patients infected with subtypes A and B in clinical characteristics, such as fever, cough, asthma, pulmonary rales, heart rate, and final diagnosis. By age stratification, we only found that in the 6–12 months of age group, patients infected with subtype A had high frequency of fever and in the 12.1–36 months of age group patients infected with subtype A had longer fever duration, which was consistent with data reported by Gilca et al. [27]. Finally, laboratory tests results, including cytokine, T-cell subsets, biochemical criterion, hematological analysis and so on, were analyzed. We found most of the data were not significantly different between subtypes A and B except for CRP, IL-10 and CD4/CD8 rate. Further analysis revealed gender-specific differences in CRP, IL-10 and CD4/CD8 rate between subtypes A and B. The differences in CRP were mainly found in males, while the differences in IL-10 and CD4/CD8 rate were mainly found in females. We found that the lymphocyte percentage was lower in subtype A than B in the 6–12 months age group, and WBC, PLT and the frequency of abnormal CD4 count was lower in subtype A than B in the 12.1–36 years age group, while the frequency of abnormal AST, IL-6 and LDH level were higher than subtype B. Therefore we concluded that the clinical severity of HRSV infection was not significantly different between subtypes A and B. There were only some differences in certain clinical variable and laboratory results; and this difference was gender-specific and age-specific. These discrepancies could be attributed to differences in study design, population and distribution of confounding factors. Some studies [43–45] have suggested that hormones and cytokines could affect CRP, IL-10 and CD4/CD8 levels, but the underlying physiological mechanisms remained unclear. Further studies with larger sample sizes were needed to confirm our results and to conclude whether there was a gender difference in the effects of HRSV A and B on CRP, IL-10 and CD4/CD8 rate. In this study, we also sought to figure out whether HRSV viral load was associated with the two subtypes and whether there was a correlation between clinical characteristics and laboratory results of each subtype. The results showed no significant difference in viral load between the two subtypes of HRSV (*P* = 0.465). However, in subtype A, the duration of wheezing was significantly correlated with viral load. This suggested that respiratory symptoms, particularly wheezing, were significantly worse in patients with subtype A with increased viral load, which may prolong the recovery duration in children. This phenomenon may become a unique marker of subtype A infections. In subtype B, viral load was negatively correlated with the age of the children, the percentage of monocytes and LDH level. By combining the correlation between viral load and age, we found a significant negative correlation between viral load and age, regardless of subtype. This suggests that HRSV is more likely to replicate and multiply in younger children and that viral load decreases with age, which is more pronounced in subtype B than A. We firstly found a significantly negative correlation of viral load with LDH level and monocyte percentage in subtype B. Most viral infections can cause significant increases in monocyte percentage and LDH level, and HRSV infection is similar. However, in subtype B, the monocyte percentage and LDH level decreased as viral load increased, which speculating that subtype B infection may lead to more serious consequences for patients in the early and proliferative stages of infection. The findings need to be further explored in subsequent studies. In the future, this phenomenon may be unique to subtype B infection and become a marker to distinguish subtype A from B infection. Therefore, we speculated that knowing the viral load of infections with HRSV may help us understand the clinical course of different subtypes of HRSV virus infections.

Our study had some limitations. First, only children with ARI during the 2016 HRSV epidemic season were studied and the sample size was not large enough. This reduced the statistical power to demonstrate differences between subgroups and to classify results. In addition, variability in specimen quality increased the variability of Ct values, which would bias the results and make it more difficult to demonstrate an association between viral load and various outcomes. Lastly, only virological testing of HRSV was performed. Although researches on other respiratory pathogens as causes of ARI are valuable, the focus of this study is HRSV infection, which is known to be a major cause of severe respiratory infections in young children.

## Conclusions

In conclusion, we found that both subtypes A and B were circulating from November 2016 to April 2017 in Zhejiang Province and multiplex One-Step qRT-PCR not only provided a rapid and effective diagnosis of HRSV infection but also allowed its typing. Moreover, HRSV is the main causative virus of bronchopneumonia in infants and children, and is also a major cause of wheezing and pulmonary rales. There were no significant differences in clinical severity of HRSV infection between subtypes A and B, except for significant gender-specific and age-specific differences in some clinical characteristics variables and laboratory results. This study was also the first to describe the relationship between HRSV viral load in different subtypes and clinical symptoms. We found that knowing the viral load of HRSV infection would help to understand the clinical features of different subtypes of HRSV infection. Prospective molecular work on HRSV in larger populations over a longer period of time could be done to better define the role of HRSV subtypes in epidemiology and associated disease severity, which may influence the development of therapies and vaccines.

## Data Availability

All data generated or analyzed during this study are included in this published article [and its supplementary information files].

## Abbreviations

HRSV: Human respiratory syncytial virus
ARI: Acute respiratory infections
LRTI: Lower respiratory tract infections
IFA: Immunofluorescence Assay
CRP: C-reactive protein
PCT: Procalcitonin
IL10: Interleukin 10
AST: Aspartate aminotransferase
IL6: Interleukin 6
LDH: Lactate dehydrogenase
PLT: Platelet
IQR: Interquartile range
SpO_2_: Oxygen saturation
WBC: White blood cell
IL2: Interleukin 2
IL4: Interleukin 4
TNF: Tumor Necrosis Factor
INF-ɣ: Interferon ɣ
ALT: Alanine aminotransferase
CK: Creatine kinase
CK-MB: Creatine kinase-MB activity
